# Type 2 Diabetes Mellitus: New Genetic Insights will Lead to New Therapeutics

**DOI:** 10.2174/138920209787847023

**Published:** 2009-04

**Authors:** M.G.M Wolfs, M.H Hofker, C Wijmenga, T.W. van Haeften

**Affiliations:** 1 Department of Pathology and Medical Biology, Medical Biology Section, Molecular Genetics, University Medical Center Groningen, University of Groningen, P.O. Box 30001, 9700 RB Groningen, The Netherlands; 2 Department of Genetics, University Medical Center Groningen, University of Groningen, P.O. Box 30001, 9700 RB Groningen, The Netherlands; 3 Department of Internal Medicine, G 02-228, University Medical Center Utrecht, P.O. Box 85500, 3508 GA Utrecht, The Netherlands

**Keywords:** Type 2 diabetes, drug targets, genetics, personalized medicine.

## Abstract

Type 2 diabetes is a disorder of dysregulated glucose homeostasis. Normal glucose homeostasis is a complex process involving several interacting mechanisms, such as insulin secretion, insulin sensitivity, glucose production, and glucose uptake. The dysregulation of one or more of these mechanisms due to environmental and/or genetic factors, can lead to a defective glucose homeostasis. Hyperglycemia is managed by augmenting insulin secretion and/or interaction with hepatic glucose production, as well as by decreasing dietary caloric intake and raising glucose metabolism through exercise. Although these interventions can delay disease progression and correct blood glucose levels, they are not able to cure the disease or stop its progression entirely. Better management of type 2 diabetes is sorely needed. Advances in genotyping techniques and the availability of large patient cohorts have made it possible to identify common genetic variants associated with type 2 diabetes through genome-wide association studies (GWAS). So far, genetic variants on 19 loci have been identified. Most of these loci contain or lie close to genes that were not previously linked to diabetes and they may thus harbor targets for new drugs. It is also hoped that further genetic studies will pave the way for predictive genetic screening. The newly discovered type 2 diabetes genes can be classified based on their presumed molecular function, and we discuss the relation between these gene classes and current treatments. We go on to consider whether the new genes provide opportunities for developing alternative drug therapies.

## INTRODUCTION

The last few decades have witnessed a dramatic increase in the prevalence of type 2 diabetes mellitus, due to changes in food intake combined with less physical exercise, a lifestyle often referred to as Western. The long-term consequences of type 2 diabetes are severe and include cardiovascular disease, retinopathy, neuropathy, nephropathy, and diabetic foot disease. While it has been estimated that worldwide around one billion people are obese, over 180 million people suffer from type 2 diabetes and this number is expected to double over the next 25 years. The number of annual deaths due to type 2 diabetes is difficult to estimate because they are often hidden under cardiovascular disease, but the WHO has estimated it to be between 1 and 3 million in 2006. However, since mortality from this disease often occurs many years after its onset, even 3 million deaths is probably an underestimate of the death toll in the near future. Although external factors such as food-intake-related obesity has attracted much attention, the genetic predisposition for diabetes is also important. While the life-time risk for type 2 diabetes in the Western world is around 10%, first-degree relatives of patients have a 20–40% risk for the disease, and concordance rates for identical twins have been estimated to be 57% or higher (up to 90%) for type 2 diabetes in male twins [1]. The endeavor to find the underlying genes was unsuccessful for many years although hundreds of genetic associations have been described, based largely on candidate genes. Unfortunately, replication was only possible for a very few variants. However, the recent advent of genome-wide association studies (GWAS) holds promise, since such studies have now uncovered a number of common genetic variants related to diabetes for which replication is also possible. These genes had not previously been linked to diabetes and these loci are therefore expected to lead to new insights into the disease mechanisms.

## GLUCOSE HOMEOSTASIS AND DIABETES

Diabetes is a state of persistent hyperglycemia, leading to irreversible damage in a number of tissues, especially the retina, the kidney glomeruli, neural tissue and blood vessels. Normally plasma glucose levels are kept within a narrow range, a process referred to as glucose homeostasis. This homeostasis is regulated in a complex way and involves several axes.

After eating a meal, a person’s plasma glucose rises temporarily, after which glucose is rapidly taken up by liver and muscle and to a lesser degree by fat tissue, due to the effects of insulin released from the pancreatic β-cells. Under these circumstances, glucose is converted into glycogen, which is then stored in the liver. When plasma glucose levels have dropped, insulin secretion gradually diminishes. In the postprandial state, a minimum level of plasma glucose is assured by glucose production in the liver, partly due to release from glycogen and partly due to new formation from precursors such as lactate and the amino acids alanine and glutamine. Insulin has a number of other effects, including lipid storage in adipocytes, and it even increases DNA replication and protein synthesis. The regulation of insulin release is complex; apart from the prevailing plasma glucose level, release from various peptides from the intestine (e.g., glucagon-like peptide 1, GLP1) after a meal and feedback mechanisms ensure adequate insulin release.

Type 2 diabetes is characterized by the combination of disturbances in insulin secretion, and an impairment of the effects of insulin, so-called insulin resistance, which is often related to obesity. Insulin resistance is caused by defects in the signaling pathways that process the insulin signal in its target tissues. It has often been stated that the disease starts with insulin resistance accompanied by raised insulin and glucose levels. At a later stage, we presume β-cells undergo further damage and apoptosis, and are not able to keep up with the demand for insulin release, which then results in higher glucose levels. At least one of the pathways involved in insulin secretion, β-cell regeneration, β-cell survival, or β-cell development are important in determining the vulnerability of the β-cell pool in insulin-resistant conditions. Studies have shown decreases in β-cell function prior to the augmentation of plasma glucose in normal glucose-tolerant first-degree relatives of type 2 diabetes subjects; in these studies these relatives had normal insulin sensitivity [2, 3]. While obesity is a major risk factor for diabetes – around 50% of obese subjects will develop type 2 diabetes at some stage (depending on the age when they became obesity) –, it should be noted that 20% of type 2 diabetes subjects are not obese. It thus seems that obesity is a major risk factor for developing type 2 diabetes, but that it is the vulnerability of the β-cell pool which determines whether obesity in fact triggers type 2 diabetes.

## GENETICS OF TYPE 2 DIABETES

Although monogenic forms of diabetes have been found (Table **1**) (reviewed in [4]), the majority of cases of type 2 diabetes do not show inheritance as a Mendelian trait, but rather as a genetically complex disorder in which genetic variants predispose individuals to develop the disease. Twin studies have revealed concordance rates of over 50 % (and reported up to 90%) for type 2 diabetes in identical twins [1], which still leaves a substantial role for environmental factors, such as excess food and limited physical activity. The rapid rise in diabetes prevalence over the last few decades strongly suggests that genetic variants involved in type 2 diabetes are interacting with environmental factors.

### Candidate-Based Association Studies

Several candidate gene association studies have been performed to identify genes involved in type 2 diabetes. These studies test some of the genetic variants in a gene that is a strong candidate for being involved in the disease. Although a few genes have been identified in this way, in general these studies have not been very successful because the results could not be replicated and the p-values for association of the genetic variants were moderate.

Obviously, the few diabetes genes that were identified and replicated in candidate-based association studies had already been linked to a diabetic phenotype, since the tested genes were mainly selected on the basis of their function and their potential relationship with diabetes. The genes identified by candidate gene approaches were found to be involved in rare, monogenic forms of diabetes. The TCF2 gene was linked to maturity-onset diabetes of the young (MODY) 5 [5], while WFS1 gene mutations led to Wolfram (or DIDMOAD) syndrome, a rare, lethal, neurodegenerative disorder that includes diabetes insipidus, diabetes mellitus, optic nerve degeneration, and inner ear deafness [6]. The KCNJ11 gene is related to permanent neonatal diabetes mellitus (PNDM), a rare form of diabetes starting before the age of 6 months [7]. Finally, mutations in PPARγ can lead to insulin resistance, hypertension, and lipodystrophy [8]. Although the molecular impact of mutations in these genes is not yet fully understood, it is likely that mutations in them will have differing effects and that mutations involved in type 2 diabetes will have a milder impact on human physiology than those involved in monogenic diabetes.

### Genome-Wide Association Studies (GWAS)

Recent advances in genotyping techniques and the collection of large, type 2 diabetes patient cohorts have made it possible to perform hypothesis-free genome-wide association studies (GWAS) to identify common genetic variants that increase susceptibility to type 2 diabetes. The human genome harbors around 3 billion base-pairs, which contain at least 3 million common single nucleotide polymorphisms (SNPs) according to the International HapMap Project. However, alleles represented by SNPs that are close together have often stayed on the same chromosome in further generations, forming a so-called haplotype. This implies that when one variant has been typed, we know the genotype of a set of other variants that surround the initial genetic marker; in genetic terminology it is said that the SNPs that stayed together on a chromosome are in high linkage disequilibrium. It has been estimated that, in a Caucasian population, assessing 500,000 SNPs will detect around 80% of the common genetic variation. GWAS typically involve the assessment of such numbers of SNPs determined in large case-control studies for association with a disease or with a so-called “phenotypic trait”, i.e., type 2 diabetes. It should be noted that GWAS identify association of a genetic locus, and not directly of a gene. It is likely that the most associated SNPs in a genomic region are not the causal variant, but that the disease-producing SNP is in high linkage disequilibrium with the “associated” SNPs. It is also possible that a SNP, even when located in a gene, can influence the expression of a nearby gene located several thousand base-pairs or more away [9]. It is therefore difficult to determine which gene is responsible for the association signal in a GWAS with full certainty. A detailed description of a GWAS is reviewed by McCarthy *et al.* [10].

The genome-wide approach has been very successful for type 2 diabetes, leading to the identification of over a dozen common genetic variants associated with the disease lying near genes that had not previously been associated with a diabetic phenotype (Table **1**) [11-24]. In the near future this number will probably increase and a large part of the genetic variation that confers susceptibility to type 2 diabetes will be unveiled. At present, the total effect of the identified genetic variants explains only a small part of the total estimated genetic variation which is mainly due to the small effect from each of the identified variants (with odds ratios around 1.2 for each gene, which implies a mere 20% extra risk for the disease) [25, 26]. Identifying more genetic variants - by including more cases to increase the statistical power of GWAS, performing GWAS in non-Caucasian populations, improving the genomic coverage of the GWAS, and performing meta-analyses of the studies published so far - could overcome these problems. Moreover, thorough analysis of the associated region by deep sequencing could lead to variants with higher odds ratios being identified (see section 5).

### Functions of Type 2 Diabetes Genes

As discussed above, type 2 diabetes is a disease characterized by impaired β-cell secretion of insulin, in combination with resistance to insulin in its target tissues. Both insulin secretion and insulin sensitivity are influenced by genetic and environmental factors [27]. Genes that harbor variants associated with diabetes can thus be expected to exert their effect through one of these pathways.

Most genes that play a role in monogenic diabetes are important for pancreatic β-cell growth and/or function (Table **1**). Genetic variants in three (KCNJ11, TCF2, WFS1) out of four replicated genes for type 2 diabetes found by candidate-based genetic association studies are also related to decreased β-cell function. The best characterized gene is KCNJ11 which encodes a member of a β-cell potassium channel involved in insulin secretion [28]. Mutations in TCF2 are associated with insulin secretion [29], while knock-out mutations in WFS1 have been found to lead to Endoplasmic Reticulum (ER) stress and subsequent apoptosis of pancreatic β-cells [30]. The protein product of the fourth gene identified by a candidate gene approach, PPARγ, is involved in adipocyte differentiation and function (reviewed in [31]). Since rare mutations in this gene lead to insulin resistance and lipodystrophy, variations in this gene are likely to contribute to type 2 diabetes susceptibility through altered insulin effects in adipose tissue.

To explore the functions of the newly discovered genes, various studies have investigated their roles in determining sub-phenotypes of type 2 diabetes, such as peripheral insulin sensitivity and β-cell insulin secretion, and the genetic variants identified in GWAS (except KCNQ1 because variants in this gene have only recently been identified) [32-36]. In general, these studies indicate that the genetic variants in the genes identified by association studies act through interference with insulin secretion and not with peripheral insulin sensitivity, and are thus similar to the genes identified through candidate gene approaches and in monogenic diabetes. However, insulin secretion and insulin sensitivity are related through complex and poorly understood mechanisms. Expression studies have revealed that most of the newly discovered type 2 diabetes genes are expressed in multiple cell types throughout the body and that the expression of some of these genes under diabetic conditions is unaltered in the pancreatic islets whereas it is different in other tissues (reviewed in [37]). Functional studies of the molecular mechanisms by which most genetic variants lead to impaired insulin secretion and the use of animal models have not yet been published but are sorely needed.

Since only a small percentage of the variability of the genetic influence on diabetes risk has been uncovered so far, it is still possible that there are mutations in genes that affect other axes of the underlying mechanisms of diabetes. It has been proposed that multiple hits in several diabetes pathways generally occur in subjects destined to develop type 2 diabetes, and that disturbances in β-cell function are ultimately decisive for the actual development of the hyperglycemic state.

### Genetic Subgroups

**Fig. (1) Classification of diabetes genes with a potential role in β-cell function.**
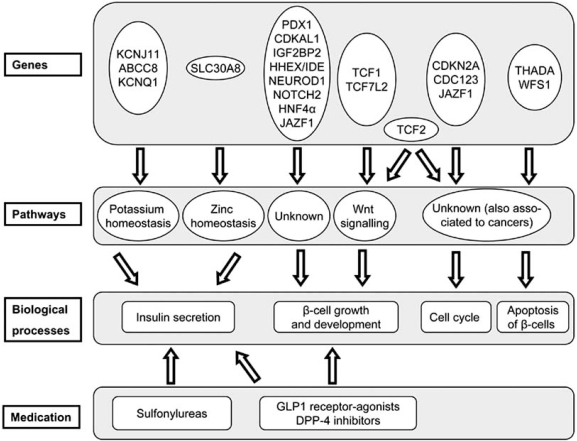
Diabetes genes involved in either type 2 diabetes, Maturity Onset Diabetes of the Young (MODY), or Permanent Neonatal Diabetes Mellitus (PNDM) are included. These genes are assigned to pathways and biological process as described in the text. Medications as shown are known to affect a subset of β-cell functions.

It can be proposed that some of the diabetes genes can be grouped into four subgroups based on what is known about their molecular function (see Fig. **1**). First, KCNJ11, KCNQ1, ABCC8, and SLC30A8 gene products are involved in cellular ion homeostasis and insulin secretion. A second group of genes – TCF7L2, TCF1, TCF2, HHEX / IDE, IGF2BP2, CDKAL1, NEUROD1, PDX1, and NOTCH2 – is likely to be involved in the growth and development of the pancreas. Of this group TCF7L2, TCF1, and TCF2 are important in Wnt signaling (reviewed in [38]). A third group is related with cell cycle events: JAZF1 and TCF2 have both been associated with prostate cancer and these genes might play a role in the regulation of the cell cycle [39-41]. CDKN2A and, presumably, CDC123 also play a role in the cell cycle. Finally, THADA and WFS1 are thought to be involved in the apoptosis of β-cells. Genes involved in the cell cycle and apoptosis of β-cells might play a role in diabetes by dysregulating the response of β-cells when a higher insulin output is called for when insulin resistance is present.

## MANAGEMENT OF TYPE 2 DIABETES AND HOW IT IS RELATED TO GENETICS

The corner stone for diabetes management still lies in diet and exercise [42, 43]. There is also a slowly expanding list of drugs being used to treat type 2 diabetes, all of which act through one of the pathways important in diabetes pathophysiology. However, neither changes in lifestyle nor the use of medication are sufficient to cure diabetes, although both interventions can delay the progression of disease. There is therefore an urgent need to develop new medications or strategies to counter the huge increase in cases expected in the future. Since the management of type 2 diabetes with either lifestyle changes, medication or both, is more effective when started at an early stage, improving the techniques for early diagnosis and the opportunities for early intervention will greatly improve the effects of current ways of managing type 2 diabetes.

Pathways important for the function, growth and development of pancreatic β-cells provide obvious drug targets and are already being used, since defects in insulin secretion play a central role in diabetes. Sulfonylurea derivatives are widely used and act through improving β-cell function by closing β-cell potassium channels and thereby enhancing insulin secretion. Some of the genes associated with both monogenic (ABCC8 and KCNJ11) and complex diabetes (KCNJ11, KCNQ1) encode subunits of these potassium channels and accordingly, monogenic diabetes of this type can be well managed with sulfonylureas. SLC30A8 encodes a zinc transporter protein in the β-cell and the function of this gene product might be related to that of the potassium channel genes by regulating ion homeostasis in the β-cell [44].

The actions of various other drugs involve provoking different effects rather than augmenting insulin secretory function: thiazolidinediones enhance insulin activity by acting on adipose tissue, metformin lowers hepatic glucose output, and pramlintidine delays gastric emptying and inhibits the release of glucagons. GLP1 receptor-agonists or DPP-4 inhibitors have combined actions on food intake by regulating satiety and enhancing insulin secretion in the short term, and β-cell neogenesis and proliferation in the long term (reviewed in [45]). The exact physiological mechanisms that underlie these improvements are, however, unclear. Except for PPARγ and genes involved in pancreas growth and survival, there is no evidence that any of the other genes identified by GWAS are involved in any of these processes. The protein product of PPARγ is likely to be involved in the mechanism targeted by thiazolidinediones because these compounds are ligands of PPARγ receptors [46]. Although it seems likely that some of the newly discovered diabetes genes act in a pathway that is targeted by GLP1 receptor-agonists or DPP-4 inhibitors, more research into both the mechanisms of the medication and the function of the genes is needed.

## IMPLICATIONS FOR PREVENTION AND TREATMENT

### Genetic Screening for Prediction and Prevention

The effectiveness of current type 2 diabetes management is greatly improved when it is started at an early stage of the disease. If genetic testing could be used to predict type 2 diabetes, preventive measures could be taken and diabetes could potentially be managed more easily. However, the variants associated with type 2 diabetes that have been identified so far only explain a small percentage of the total genetic variation that is thought to be present [25, 26]. It is therefore not yet possible to perform accurate predictive genetic testing but, in the near future, research should provide more insight into the opportunities for such testing. Firstly, it is expected that many more common genetic variants will be identified by performing GWAS in other populations and by improving their power and genomic coverage (i.e., more patients included in studies and more SNPs tested). Secondly, performing thorough analyses of the genomic regions that show association to the disease by resequencing large numbers of patients and controls may identify genetic variants that have higher odds ratios than the common genetic variants identified so far. In a GWAS it is unlikely that the functional variant will be identified, but rather one or more common variants to which the disease variant is in linkage disequilibrium, i.e., that the actual causative mutation(s) is in close vicinity to the tested variant. If the frequency of the causal disease variant is lower than the tested common SNP, we can expect the odds ratio of the causal SNP to be higher and this would make the causal SNP a better candidate to use for genetic testing. One drawback of genetic testing is that many variants have to be tested for each subject, which is laborious and costly at present, but improved genotyping and resequencing technologies will make screening for such variants feasible in the near future.

### New Opportunities for Intervention

Even if the exact functions of the majority of the genes associated with type 2 diabetes are still elusive, they can be broadly grouped into several classes (see Fig. **1**). When certain genes are involved in the same molecular pathway or physiological process, not only these genes but the entire pathway or process would be an obvious target for new anti-diabetes drugs. In combination with genetic screening, such information could be used to optimize diabetes management by prescribing drugs that act on those pathways that are affected in a patient, and *vice versa*, it is also possible that altering the activity, be it stimulation or inhibition, of undisturbed processes could improve glucose homeostasis in selected patients. Improved drug treatment in diabetes can be seen in the current management of various MODY subtypes. As described above, mutations in genes involved in β-cell signaling and/or growth in the pancreas have been found to be responsible for MODY subtypes. After these mutations were identified, the management of these diseases was greatly improved by the use of sulfonylurea derivatives, which enhances insulin secretion instead of exogenous insulin [44]. The good response of MODY patients to sulfonylurea treatment serves as an excellent example of personalized therapy based on genetic screening.

Evidence that genes identified by GWAS can represent targets for managing type 2 diabetes is provided by the association to this disease of variants in the KCNQ1 and KCNJ11 genes. Both these genes encode proteins that are members of β-cell potassium channels important for insulin secretion and these channels are targeted by sulfonylurea derivates which are already widely used anti-diabetic drugs [44].

Another gene, found through a candidate-based association study and likely to act in the pathway targeted by thiazolidinediones, is PPARγ. On the physiological level no relationships are known between current medications and all the other genetic variants identified in either GWAS or candidate gene association studies. Therefore all these genes represent new potential targets for intervention.

Type 2 diabetes genes that are presumed to play a role in cell cycle regulation (JAZF1, CDKN2A, and CDC123), apoptosis (THADA and WFS1), or pancreas development and growth (TCF7L2, TCF2, HHEX / IDE, IGF2BP2, CDKAL1, and NOTCH2) have not yet been studied well enough to predict whether interference with their products could be used in managing diabetes. Studies are needed in order to better classify these genes.

Common variants in type 2 diabetes genes relating to cell cycle events and apoptosis, and representing different alleles than those associated to type 2 diabetes, are also associated with various cancers. In addition, the risks of developing diabetes and prostate cancer are correlated in a complex way: overall diabetes risk and prostate cancer risk are inversely correlated, but while diabetes risk and the risk of developing aggressive prostate cancer show no correlation, these two risks *are* positively correlated in lean men and in men who undertake vigorous physical activity [47]. Systemic administration of agents interfering with the gene products of diabetes genes that are also associated with cancers could, therefore, be beneficial in treating diabetes, but they might be potentially carcinogenic. Tissue-specific drug targeting or additive medication to overcome the carcinogenic side-effects of such medication needs to be developed to overcome this problem. Hence, cell-cycle related genes are probably not the most promising targets for developing new diabetes treatments, unless cell-type-specific targeting of drugs becomes a reality.

Although the genes containing variants associated with type 2 diabetes that seem to be involved in pancreatic growth and development have not been well studied yet, we can speculate on several possibilities for intervention with such genes. Because most genes in this category are not associated with other diseases, they represent promising intervention targets. Variants in these genes might affect the regenerative or proliferative potential of the β-cell population when there is an increased demand for insulin secretion. In this case it would be possible to use such genes, or the molecular pathways they are involved in, as a target for intervention, in an attempt to correct the poor response of the β-cells and ultimately aiming to improve insulin secretion.

Another possibility is that variants in these genes cause pancreatic damage, which would lead to endocrine malfunction of the pancreas at an early stage of the diabetic development. The possibilities for therapeutic intervention would largely depend on the severity and reversibility of such a malfunction.

Genetic variants in three genes identified (or confirmed) by GWAS (TCF7L2, CDKAL1, and SLC30A8) are associated with impairment of proinsulin-to-insulin conversion, whereas variants in other genes identified by GWAS are not associated to this [48]. Even if the affected proinsulin-to-insulin conversion is not a primary but a secondary effect of genetic variants in one of the three genes, reconstituting this process would enhance insulin secretion and thereby enhance β-cell function. Although the conversion of proinsulin-to-insulin has long been known to be involved in type 2 diabetes [49], the mechanisms that regulate this process are still undetermined and there is no current therapy that acts by interfering with this process. Association of TCF7L2, CDKAL1, and SLC30A8 to impaired proinsulin-to-insulin conversion has provided evidence that these genes are somehow related to this process and this clears the way for functional research and development of new medication.

## CONCLUSIONS

GWAS are an important tool for identifying genetic variation and they have been very successful in finding 19 genetic loci involved in type 2 diabetes. Genotyping techniques will continue to improve and patient cohorts will become larger, which will lead to more genetic variants being identified in the near future. Although the true causal SNPs are often not known, it is anticipated they will be uncovered by deep sequencing of the genomic regions containing association signals. These advances will help elucidate a higher percentage of the total genetic variation, and improve opportunities for genetic screening and personalized diabetes care. Functional studies and animal models harboring specific gene deletions will be needed to study the role of such mutations, while genetic studies using sharply defined endophenotypes will greatly enhance our knowledge about the type 2 diabetes genes identified by GWAS.

At present, it is too early to expect results from GWAS to lead to new therapies. Nevertheless, it is clear that genetic studies are crucial to dissecting the mechanisms underlying the biological processes and to finding ways to intervene with them, as they provide important clues for directing the focus of functional research. The ongoing avalanche of genetic information about type 2 diabetes will thus pave the way to further research in the field and has already yielded important information to aid the development of new therapeutic strategies.

## Figures and Tables

**Table 1.** Genes Associated with Diabetes: Overview of their Target Tissue^1^, Function^2^, and Related Medication^3^

Diabetes Gene	Proposed Diabetes Target Cell Type / Tissue^1^	Monogenic Diabetes^4^	Type 2 Diabetes^5^	Proposed Function(s) for Gene Product^2^	Drug(s) Affecting the Same Pathway as the Diabetes Gene^3^
ABCC8	Pancreas β-Cell	X		- B-cell ion homeostasis and insulin secretion; ATP-binding cassette transporter that modulates ATP-sensitive potassium channels and insulin release	Sulfonylurea derivatives
ADAMTS9	Unknown		X	- Cleavage of proteoglycans	Unknown
CDC123	Pancreas β-Cell		X	- Cell cycle regulation	Unknown
CDKAL1	Pancreas β-Cell		X	- Growth and development - Proinsulin to insulin conversion	Unknown
CDKN2A	Pancreas β-Cell		X	- Cell cycle regulation	Unknown
CEL	Unknown	X		- Glycoprotein that is important in regulation of cholesterol metabolism	Unknown
FTO	Hypothalamus		X	- Associated to obesity	Unknown
GCK	Unknown	X		- Catalyzes reaction from glucose to glucose-6-phosphate	Unknown
HHEX	Pancreas β-Cell		X	- Growth and development; transcription factor	Unknown
HNF4α	Pancreas β-Cell	X		- Growth and development; transcription factor	Unknown
IDE	Pancreas β-Cell		X	- Termination of the response to insulin	Unknown
IGF2BP2	Pancreas β-Cell		X	- Growth and development	Unknown
JAZF1	Pancreas β-Cell		X	- Cell cycle regulation; transcriptional repressor	Unknown
KCNJ11	Pancreas β-Cell	X	X	- B-cell ion homeostasis and insulin secretion	Sulfonylurea derivatives
KCNQ1	Pancreas β-Cell		X	- B-cell ion homeostasis and insulin secretion	Sulfonylurea derivatives
KLF11	Unknown	X		- Unknown	Unknown
NEUROD1	Pancreas β-Cell	X		- Growth and development; transcription factor that activates several genes including insulin and is important for early β-cell development	Unknown
NOTCH2	Pancreas β-Cell		X	- Growth and development; transcription factor; receptor for membrane bound ligands	Unknown
PDX1	Pancreas β-Cell	X		- Growth and development; nuclear protein that acts as a transcriptional activator of several genes including insulin and is important for early β-cell development	Unknown
PPARG	Adipocyte		X	- Nuclear receptor (transcription factor) that regulates adipocyte differentiation	Thiazolidinediones
SLC30A8	Pancreas β-Cell		X	- B-cell ion homeostasis and insulin secretion; cellular efflux of Zn2+ ions - Proinsulin to insulin conversion	Sulfonylurea derivatives
TCF1	Pancreas β-Cell	X		- Growth and development; Transcription factor that forms a complex with the product of TCF2 important for Wnt signaling	Unknown
TCF2	Pancreas β-Cell	X	X	- Growth and development; transcription factor that forms a complex with the product of TCF1 important for Wnt signaling - Cell cycle regulation	Unknown
TCF7L2	Pancreas β-Cell		X	- Wnt signaling - Proinsulin to insulin conversion	Unknown
THADA	Pancreas β-Cell		X	- Apoptosis	Unknown
TSPAN8	Unknown		X	- Glycoprotein involved in the mediation of signal transduction	Unknown
WFS1	Pancreas β-Cell	X	X	- Apoptosis; Endoplasmic Reticulum stress pathway activation	Unknown

Genes included in the list are involved in type 2 diabetes, Maturity Onset Diabetes of the Young (MODY), or Permanent Neonatal Diabetes Mellitus (PNDM). The cut-off p-value for the inclusion of type 2 diabetes genes identified by GWAS is 1 x 10^-8^ [12, 21-23]. The third and fourth columns of the table show whether a gene is involved in monogenic^4^ or complexly inherited type 2 diabetes^5^.
